# Editorial: Interaction of endocrine disorders and metabolic associated fatty liver disease: the genetic and epigenetic basis

**DOI:** 10.3389/fendo.2023.1308860

**Published:** 2023-11-07

**Authors:** Qin Pan, Han-Qing Chen, Wan-Cheng Chow, Hui-Ping Zhou

**Affiliations:** ^1^ Research Center, Shanghai University of Medicine & Health Sciences Affiliated Zhoupu Hospital, Shanghai, China; ^2^ Guangzhou First People’s Hospital, School of Medicine, South China University of Technology, Guangzhou, China; ^3^ Department of Gastroenterology and Hepatology, Singapore General Hospital, Singapore, Singapore; ^4^ Department of Microbiology and Immunology and McGuire Veterans Affairs Medical Center, Virginia Commonwealth University, Richmond, VA, United States

**Keywords:** metabolic associated fatty liver disease, metabolism, steatosis, inflammation, fibrosis, genetics, epigenetics

In contrast to non-alcoholic fatty liver disease (NAFLD) and alcoholic fatty liver disease (ALD), metabolic-associated fatty liver disease (MAFLD) reflects a novel classification of chronic liver disease with a proposed definition of hepatic steatosis and one of the three criteria, namely, overweight/obesity, type 2 diabetes mellitus, metabolic dysregulation (1). Clinical evidence has presented excellent concordance between the diagnosis of MAFLD and NAFLD (2), which features hepatic steatosis, ballooning, lobular inflammation, liver fibrosis/cirrhosis, and may result in hepatocellular carcinoma (HCC). Furthermore, recent exploration of this issue increases our knowledge about the epidemiological, genetic, and epigenetic basis underlying MAFLD and related pathological characteristics.

In addition to alcohol abuse, hepatic steatosis resulting from a Western diet and sedentary lifestyle is now recognized to be an important component of metabolic syndrome (MetS). It causatively predisposes individuals to multiple MetS components and other endocrine-based metabolic disorders by, to a large extent, complicated inter-organ communications. The prevalence of pancreatic steatosis and iron overload have been assessed by MRI-biomarkers of proton density fat-fraction (PDFF) and iron accumulation, respectively, and found by Marti-Aguado et al. to increase with the number of metabolic traits. Pancreatic PDFF also increases with the grade of hepatic steatosis and non-alcoholic steatohepatitis (NASH) diagnosis. Moreover, both indexes independently associate with a high risk of cardiovascular disease in patients with NAFLD.

Adipokines, cytokines, and metabolites usually play a mediative and/or prospective role in the interaction between endocrine disorders and MAFLD. Zhang et al. highlight significantly lower levels of circulating omentin in patients with MAFLD, especially in the Asian population, when compared to healthy controls. The decreased circulating omentin levels are further associated with MetS as well as fasting blood glucose (FBG), obesity, carotid atherosclerosis, and hypertension. Another meta-analysis uncovers the lowered omentin levels in patients with type 2 diabetes mellitus (T2DM) in comparison to those in the control group. Consistent with its protective action during various pathophysiological processes (inflammation, oxidation, and apoptosis), the lack of adipocyte-derived omentin upregulates the risk of complications in patients with T2DM.

The real-world study by Ma et al. shows that the second to highest quartiles of blood lactate levels are independently associated with increasing risk of MAFLD in patients with T2DM, both in those who are undergoingmetformin treatment and those who are not.

Interestingly, genetic polymorphism may exert different, even opposite, effects on hepatic and extrahepatic metabolic characteristics. Despite some controversy over its actions, the T allele at rs641738 of membrane-bound O-acyltransferase domain containing 7 (MBOAT7) has been reported by Xu et al. to confer a high risk of NAFLD in individuals of European descent. However, the TT genotype at MBOAT7 rs641738 is associated with reduced risk of MetS and T2DM in the MAFLD population. In addition, the TT genotype demonstrates an intimate association with a lowered risk of obesity and atherosclerotic cardiovascular disease (ASCVD) in the cohort of elderly Chinese. Similar observations can be obtained at PNPLA3 rs1010023. C-allele at PNPLA3 rs1010023 (CC and TC genotypes) sensitizes chronic hepatitis B (CHB) patients to hepatic steatosis, and brings about significant improvement in the homeostasis model assessment index (HOMA-IR) and FBG. The polymorphism-based redistribution of visceral and subcutaneous lipids could provide us with a rational, yet incomplete, explanation.

‘Multiple hits’ in the condition of hepatocellular steatosis, such as lipid peroxidation, endoplasmic reticulum stress, injury, and programmed cell death, stimulate the progression of lobular inflammation. Huang et al. describe that impaired mucosal barrier, LPS leakage, toll-like receptor 4 (TLR4), and TLR7 signaling occur upon dysbiosis and then activate the resident macrophages in the liver (Kupffer cells) to induce an innate immune response. In addition, activated TLR7 signaling in Kupffer cells promotes NASH by hepatocyte death and Treg inhibition. These inflammatory responses lead to an activation of hepatic stellate cells (HSCs), whereas M1 polarization of liver macrophages activates HSCs by the cGAS-STING pathway. The HSC activation resultantly gives rise to liver fibrosis/cirrhosis. In male NAFLD patients with the MBOAT7 rs641738 (C>T) variant, the predisposition of hepatic steatosis is accompanied by deteriorated indexes of hepatocyte impairment (ALT) and liver fibrosis (stiffness).

MAFLD has now been revealed to correlate with HCC, partially on the basis of chronic liver inflammation through pattern recognition receptors (*i.e*., TLRs). The present Research Topic highlights the promotive role of TLR4 in the development and metastatic potential of steatohepatitis-related HCC. Contrastively, induction of pattern recognition receptors is critical for systemic anti-tumor immunity *via* the release of type I interferon and maturation of dendritic cells. Genetic factors, such asmutations and polymorphisms, and epigenetic dysregulation (*i.e*., DNA methylation, histone modification, and non-coding RNAs) have been well described to be involved in these processes.

In summary, the liver takes the central place in an inter-organ network. Reciprocal causation is suggested between hepatic and extrahepatic metabolic disorders, with outcomes of hepatic steatosis, inflammation, fibrosis, and HCC. Various metabolic disorders may share a genetic or epigenetic basis, sometimes with different patterns.

## Author contributions

QP: Writing – original draft; Writing – review & editing. HQC: Writing – review & editing. WCC: Writing – review & editing. HPZ: Writing – review & editing.
